# Microglial activation decreases retention of the protease inhibitor saquinavir: implications for HIV treatment

**DOI:** 10.1186/1742-2094-10-58

**Published:** 2013-05-04

**Authors:** Shannon Dallas, Michelle L Block, Deborah M Thompson, Marcelo G Bonini, Patrick T Ronaldson, Reina Bendayan, David S Miller

**Affiliations:** 1 National Institute of Environmental Health Sciences, National Institutes of Health, Research Triangle Park, NC, USA; 2 Drug Metabolism and Pharmacokinetics, Drug Safety Sciences, Janssen R&D, LLC, Spring House, PA, USA; 3 Department of Anatomy and Neurobiology, Virginia Commonwealth University Medical Campus, Richmond, VA, USA; 4 Science and Technology Development, North Carolina Biotechnology Center, Research Triangle Park, NC, USA; 5 Section of Cardiology and Department of Pharmacology, College of Medicine, University of Illinois at Chicago, Chicago, IL, USA; 6 Department of Pharmaceutical Sciences, Faculty of Pharmacy, University of Toronto, Toronto, ON, Canada; 7 Department of Medical Pharmacology, College of Medicine, University of Arizona, Tucson, AZ, USA

**Keywords:** Drug transporters, HIV, Inflammation, MEK1/2, Microglia, Multidrug resistance proteins, NF-kappa β, P-glycoprotein, Saquinavir, Solute carrier uptake transporters, Toll-like receptor

## Abstract

**Background:**

Active HIV infection within the central nervous system (CNS) is confined primarily to microglia. The glial cell compartment acts as a viral reservoir behind the blood-brain barrier. It provides an additional roadblock to effective pharmacological treatment via expression of multiple drug efflux transporters, including P-glycoprotein. HIV/AIDS patients frequently suffer bacterial and viral co-infections, leading to deregulation of glial cell function and release of pro-inflammatory mediators including cytokines, chemokines, and nitric oxide.

**Methods:**

To better define the role of inflammation in decreased HIV drug accumulation into CNS targets, accumulation of the antiretroviral saquinavir was examined in purified cultures of rodent microglia exposed to the prototypical inflammatory mediator lipopolysaccharide (LPS).

**Results:**

[^3^H]-Saquinavir accumulation by microglia was rapid, and was increased up to two-fold in the presence of the specific P-glycoprotein inhibitor, PSC833. After six or 24 hours of exposure to 10 ng/ml LPS, saquinavir accumulation was decreased by up to 45%. LPS did not directly inhibit saquinavir transport, and did not affect P-glycoprotein protein expression. LPS exposure did not alter RNA and/or protein expression of other transporters including multidrug resistance-associated protein 1 and several solute carrier uptake transporters.

**Conclusions:**

The decrease in saquinavir accumulation in microglia following treatment with LPS is likely multi-factorial, since drug accumulation was attenuated by inhibitors of NF-κβ and the MEK1/2 pathway in the microglia cell line HAPI, and in primary microglia cultures from toll-like receptor 4 deficient mice. These data provide new pharmacological insights into why microglia act as a difficult-to-treat viral sanctuary site.

## Introduction

Microglia are increasingly implicated in the pathogenesis of numerous neurodegenerative disorders including Parkinson’s, Alzheimer’s, and lateral sclerosis [1]. The evidence for a microglial component in the development of dementia is particularly convincing in HIV/AIDS patients, as the primary site of CNS infection is within resident microglia cells. Once infected, activated microglia release a number of pro-inflammatory mediators that directly damage nearby neurons, and deregulate normal brain parenchymal homeostasis through their interaction with astrocytes and uninfected microglia [2]. Despite recent advances in antiretroviral (AR) therapies, HIV-related neurological symptoms remain difficult to treat and HIV-associated neurological impairment continues to be very high among patients who are now living with a ‘chronic’ disorder [3].

One limitation of current HIV/AIDS drug regimens continues to be the development of ‘pharmacological resistance’ to currently available AR drugs. That is, high levels of energy-dependent efflux drug transporters such as P-glycoprotein and multidrug resistance-associated proteins (MRPs) in target cells prevent accumulation of drugs to levels sufficient to provide a therapeutic effect [4]. Clinically, lower amounts of protease inhibitors such as saquinavir and ritonavir accumulate in peripheral viral compartments such as blood mononuclear cells in HIV-infected patients who demonstrate greater P-glycoprotein and MRP1 expression [5,6]. Using P-glycoprotein knock-out mice, multiple laboratories have confirmed that P-glycoprotein significantly limits brain accumulation of saquinavir, and other protease inhibitors including indinavir and amprenavir at the level of the blood-brain barrier (BBB) [7-10]. Previously, we have demonstrated that cultured rat microglia also express multiple drug transporters including P-glycoprotein [11], Mrp1 [12], and Mrp4/5 [13]. These transporters are not only present in significant quantities but are also functionally active, able to transport a variety of known anti-HIV medications including zidovudine, saquinavir, ritonavir, indinavir and atazanavir [14-16].

In addition to HIV, AIDS patients frequently suffer multiple bacterial and viral co-infections (for example, CNS toxoplasmosis, cryptococcosis, and tuberculosis) and are under a constant state of generalized brain inflammation. This leads to deregulation of microglial cell function and release of pro-inflammatory mediators (for example, IL1β; TNF-α), reactive oxygen species, and viral proteins (gp120; tat), all shown to alter transporter expression and/or function in multiple cell types via complex, and often redundant, signaling pathways [17-20]. In brain endothelial cells, TNF-α induces Mdr1b promoter activity via nuclear translocation of the transcription factor NF-κβ [21]. Similarly, changes in P-glycoprotein transport activity and expression in isolated rat brain capillaries occurs following activation of the toll-like receptor 4 (TLR4), which in turn triggers a cascade of molecular signaling events involving TNF-α, endothelin-1, nitric oxide synthase and protein kinase C [22]. More recently, Mrp1 expression in primary rat astrocytes was also shown to be regulated by TNF-α (but not IL-6 or IL-1β), via NF-κβ and c-Jun N-terminal kinase signaling [23]. How then, a self-propelling cycle of inflammation and consequent cellular dysfunction affects expression and function of microglial drug transporters, and how this might ultimately affect brain AR drug exposure is unknown. To begin to answer these questions we have examined how drug transport is altered in microglial cells following treatment with the prototypical inflammatory mediator lipopolysacharride (LPS). We show that LPS exposure reduces cellular accumulation of the protease inhibitor saquinavir and examine possible mechanisms underlying this effect.

## Methods and materials

### Materials

Saquinavir was kindly provided from Roche Products Inc. (Hertfordshire, UK). [^3^H]saquinavir (58 Ci/mmol) was synthesized by Amersham (Piscataway, NJ, USA). MK571 was obtained from Biomol (Plymouth Meeting, PA, USA). The monoclonal antibodies MRPr1 and C219 were purchased from Kamiya Biomedical (Seattle, WA, USA), and Signet (Dedham, MA, USA), respectively. 2-(N,N-diethylamino)-diazenolate-2-oxide (DEA NONOate) was purchased from Alexis Biochemicals (Plymouth Meeting, PA, USA). [N-(3-Aminomethyl)benzylacetamidine] (1400W), bisindolylmaleimide I (BIM), 1,4-Diamino-2,3-dicyano-1,4-bis(2-aminophenylthio)butadiene (U0126), diphenyleneiodonium (DPI), endothelin-1 (ET-1), [4-(4-Fluorophenyl)-2-(4-methylsulfinylphenyl)-5-(4-pyridyl)1H-imidadole] (SB203580); 4-hydroxy-2,2,6,6-tetramethylpiperidene-1-oxyl (Tempol), N^G^-monomehtyl-L-arginine monoacetate (L-NMMA), interleukin1beta (IL-1β), LPS (*E.Coli* 0111:B4); phorbol-12-myristate-13-acetate (PMA), 5-pregnen-3β-ol-20-one-16α-carbonitrile (PCN), prostaglandin E_2_ (PGE_2_), 1,9-pyrazoloanthrone (JNK II), NF-κβ inhibitor peptide (SN50), tissue inhibitor of metalloproteinase-3 (TIMP3), tumor necrosis factor-alpha (TNF-α), type I IL-1β receptor antagonist (IL-1β RA), wortmannin, and antibodies against IL1-β and TNF-α were all purchased from Calbiochem (La Jolla, CA, USA). Fucoidan was obtained from Sigma (St Louis, MO, USA). All tissue culture reagents were purchased from Invitrogen (Carlsbad, CA, USA) unless otherwise indicated.

### Animals

Timed pregnant (gestation E14) Wistar and Fisher rats were purchased from Charles River Laboratories (Wilmington, MA, USA). Timed Pregnant (gestation E14) C3HeB/FeJ (TLR4^+/+^), and C3H/HeJ (TLR4^-/-^) mice were purchased from The Jackson Laboratory (Bar Harbor, ME, USA). The C3H/HeJ strain contains a spontaneous mutation in the TLR4 gene making these mice deficient in TLR4-mediated responses, where they are resistant to endotoxins such as LPS. All animals were maintained in a strict pathogen-free environment. All studies were approved by the National Institutes of Environmental Health Sciences institutional review board and adhered to NIH guidelines for the care and handling of experimental animals.

### Primary cultures of microglia

Primary microglia-enriched cultures were prepared from whole brains of one to two day-old mice and rats as described previously, with modifications [24]. Following decapitation, whole brains were removed, and brain tissue triturated after meninges and blood vessel removal. Cells (5×10^7^) were seeded in complete medium (DMEM containing 10% fetal bovine serum, penicillin (50 U/ml)/streptomycin (50 mg/ml), L-glutamine (2 mM), non-essential amino acids (100 μM), and sodium pyruvate (1 mM), pH 7.2), in 175 cm^2^ culture flasks pre-coated with Poly-D-lysine (Sigma-Aldrich, St Louis, MO, USA). Medium was changed at 24 hours and on day seven. After 14 days, a confluent monolayer of mixed glial cells was obtained with microglia lightly adhered to the astrocyte layer. Essentially pure microglia cultures (> 98% as determined by IBA-1 staining) were then obtained from shaking the lightly adherent microglia, and seeding the cells in 24-well plates for subsequent assays. Cells were used for subsequent experiments at 24 hours post-shaking.

### Microglia cell line

The continuous rat microglia cell line HAPI was originally isolated from mixed glial cultures prepared from three day-old rat pups, and was a generous gift of Dr James R Connor (Hershey Medical Center, Pennsylvania State University, Hershey, PA, USA). The cells exhibit prototypical microglia type behavior including the ability to phagocytose, and to release TNF-α and NO upon stimulation with LPS [25]. The cell line was maintained at 37°C in DMEM supplemented with 10% FBS, 50 U/mL penicillin and 50 μg/mL streptomycin in a humidified incubator with 5% CO_2_/95% air. Cells were passaged twice weekly using 0.25% trypsin containing EDTA. Passages six through twelve were used for all studies.

### Microglial incubation with LPS and signal transduction activators and inhibitors

Primary cultures of microglia and HAPI cells were plated in 2-well dishes for transport, nitrite and TNF-α assays or 25 cm^2^ flasks for immunoblotting and PCR assays, and incubated with 1 to 10 ng/ml LPS for 6 or 24 hours in MEM containing 2% FBS. Similar to LPS, the effects of various well-characterized inflammatory mediators/activators on saquinavir accumulation were examined. In this system, a decrease in saquinavir accumulation can represent either a decrease in the uptake of the compound, or an increase in the efflux of the compound. Concentrations and duration of treatment for the various pathway activators and inhibitors were consistent with previously published studies undertaken in microglia (and/or other cell types such as macrophages and brain capillaries), or based on manufacturer’s recommendations [1,22,26-28]. None of the activators or inhibitors tested in the presence or absence of LPS showed significant toxicity, as measured by the MTT assay. The following activators were tested: adenylate cyclase regulator PGE_2_ (1 μM), cytokines TNF-α (10 ng/ml) and IL-1β (10 ng/ml); the nitric oxide donor DEA NONOate (1, 10, 25 μM); rat PXR nuclear hormone receptor activator PCN (1 μM), protein kinase C activator PMA (1 μM), and the thromboxane A_2_ activator ET-1 (1 μM). For studies examining signal transduction pathway inhibition, cells were pre-incubated with pathway specific inhibitors for 30 minutes prior to the addition of LPS (10 ng/ml). Inhibitors examined were: the scavenger receptor inhibitor fucoidan (50 ng/ml); free radical scavenger Tempol (20 μM); an IL-1β receptor antagonist (10 μg/ml); c-Jun N-terminal kinase inhibitor JNK II (10 μM); MAP kinase (MEK)-1 and -2 inhibitor, U0126 (10 μM); NADPH oxidase inhibitor DPI (10 nM); nitric oxide synthase inhibitor 1400W (5 μM); NF-κβ peptide inhibitor, SN50 (10 μM); p38 MAP kinase inhibitor SB203580 (10 μM); phosphatidylinositol 3-kinase (PI3-kinase) inhibitor wortmannin (1 μM); protein kinase C inhibitor BIM (25 nM); metalloproteinase inhibitor TIMP3 (10 ng/ml); and antibodies against TNF-α (0.5 μg/ml), IL-1β (0.5 μg/ml), toll-like receptor 2 (anti-TLR2, 1 μg/ml) and toll-like receptor 4 (anti-TLR4, 1 μg/ml). At the conclusion of the incubation period with either the activation or inhibition compounds, cells were immediately assayed for transport, nitrite, TNF-α or protein content, as described in subsequent sections.

### [^3^H]saquinavir transport studies

Accumulation of [^3^H]saquinavir (50 nM) was measured in treated and untreated primary cultures of microglia and HAPI cells as described previously, with modifications [14]. At the conclusion of the pathway activator/inhibitor incubation, cells were washed once and pre-conditioned for 30 minutes at 37°C with transport medium (Earle’s balanced salt solution (EBSS), containing 1.8 mM CaCl_2_, 5.4 mM KCl, 0.8 mM MgSO_4_, 138 mM NaCl, 1.0 mM Na_2_HPO_4_, 5.5 mM D-glucose and 20 mM HEPES, pH 7.4). Cells were then incubated for the desired time with transport medium containing [^3^H]saquinavir (50 nM) with or without various transport inhibitors. At completion of the accumulation period, the transport medium was removed by aspiration and accumulation was terminated by adding ice-cold EBSS. Cells were solubilized for 30 minutes with 1 N NaOH and then transferred to scintillation vials containing 2 N HCl and scintillation cocktail (Beckman Coulter; Fuller, CA, USA). Cellular incorporation of the radiolabeled probe was measured using liquid scintillation counting. The sample counts in each well were standardized to the amount of cell protein (mg/ml) present, as determined by the Bradford colorimetric method (Bio-Rad, Hercules, CA, USA) with BSA (Sigma-Aldrich, St Louis, MO, USA) as the standard.

### Nitrite measurement

Nitric oxide production and release by primary cultures of microglia and HAPI cells were determined by measurement of nitrite levels using a colorimetric method with Griess reagent (0.1% *N*-[1-naphthyl] ethylenediamine dihydrochloride, 1% sulfanilamide, and 2.5% H_3_PO_4_) as described previously [24]. Briefly, cells were seeded in 24-multiwell plates and incubated with and without LPS and/or various signal pathway inhibitors/activators for 24 hours. At the end of the incubation period, culture supernatants were mixed with equal volumes of Griess reagent, incubated in the dark for 10 minutes and the absorbance (540 nm) was measured with a UV MAX kinetic microplate reader (Molecular Devices, Sunnyvale, CA, USA). The absolute nitrite concentrations were determined from an eight-point sodium nitrite standard curve. The lower limit of detection of the assay was approximately 1.5 μM.

### TNF-α determination

Cells were seeded in 24-multiwell plates and incubated with and without LPS and/or various signal pathway inhibitors/activators for 24 hours. At the end of the incubation period, the production and release of TNF-α into the culture medium by primary cultures of microglia or HAPI cells was measured with a commercially available enzyme-linked immunosorbent assay (ELISA) kit from R&D Systems (Minneapolis, MN, USA), according to the manufacturer’s instructions. The absorbance (450 nm) was measured with a UV MAX kinetic microplate reader (Molecular Devices, Sunnyvale, CA, USA), using a 570 nm wavelength correction.

### RT-PCR analysis

RNA expression of several transporters was measured by reverse transcriptase polymerase chain reaction (RT-PCR). RNA (3 μg) from microglia (HAPI) was dried down, and resuspended in 5 μL of RNAse-free water. The RNA was reverse transcribed from an oligo-dT primer using Omniscript (Qiagen, Valencia, CA, USA) according to the manufacturer’s instructions. Following reverse transcription, 1.5 μL of the 20 μL reaction was used in a polymerase chain reaction (1X reaction buffer (Promega, Madison, WI, USA), 0.2 mM dNTPs, 0.25 μM each primer (Table 1), 0.25 μL Taq polymerase (Promega, Madison, WI, USA). Cycling parameters were one cycle at 95°C for five minutes, followed by 35 cycles of 94°C (20 seconds), 55°C (30 seconds), 72°C (30 seconds), and a final extension at 72°C for seven minutes. RT-PCR products were analyzed by agarose gel electrophoresis. GAPDH was used as an endogenous control. Primers for GAPDH were acquired from PE Applied Biosystems (Foster City, CA, USA).

**Table 1:** PCR Primers

**Gene**	**Forward Primer**	**Reverse Primer**
Slc22a1	TCTGGCTACAGGAGAACGACGGCCA	TGCTCCATTATCCTGACAGCTCGCGT
Slc22a2	TCGAAACAGAGGAGGATTGTGGGAATA	GAGAGGTGTTTCCCATTGCACTTAGCCAC
Slc22a6	TATGTGGGCACCTTGATTGGC	AGGGTGAGGTCCAGCCTTCCT
Slc22a8	ATCTCATCAACATCTATTGGG	AGGGCCTTTGAGTATTTTCCA
Slco1a1	TATGGAGGACAAGCCAGAGAG	AGAGGTGGTTAATCCAGCAAC
Slco1a2	CACAGAAAAAAGGCCAAGGAA	TAAGGAAAGACAGAAGGTACT
Slco1a5	CACAGAGAAAAAGCCAAAGAG	AGAGGCAGTTAAGCCGGCAAC

Slc22a, solute carrier family 22; Slco, solute carrier organic anion family.

### Immunoblotting

Crude membrane proteins were obtained from treated and untreated HAPI microglia. Following treatment, cells were washed three times with ice-cold Hanks balanced salt solution (HBSS) buffer and lysed for 10 minutes at 4°C in Cell Lytic™ M mammalian cell lysis/extraction reagent (Sigma-Aldrich, St Louis, MO, USA) containing Complete protease inhibitor cocktail (Roche, Indianapolis, IN, USA). The cell lysate was homogenized using a hand-held polytron, and the resulting cell suspension was centrifuged for 10 minutes at 4°C (1,000x*g*) to remove cellular debris. The supernatant was collected and centrifuged at 20,000x*g* for 30 minutes, followed by 100,000xg for an additional one hour. The resulting membrane pellet was resuspended in suspension buffer and frozen at −80°C until use.

For P-glycoprotein studies, crude membrane samples were separated on NuPage 7% sodium-acetate gels (Invitrogen, Grand Island, NY, USA) using a Bio-Rad minigel system (Bio-Rad, Hercules, CA, USA), and transferred electrophoretically to polyvinylidene difluoride (PVDF) membranes. The membranes were blocked for at least one hour and incubated overnight with the P-glycoprotein antibody C219 (1:100) in SuperBlock blocking buffer in TBS containing 0.5% Surfact-Amps 20 (TBS-T) (Pierce, Rockford, IL, USA) at 4°C. Following three washes (five minutes each) with TBS-T, the membranes were incubated at room temperature for two hours in the presence of anti-mouse (1:5,000) horseradish peroxidase-linked secondary antibody (Sigma-Aldrich, St. Louis, MO, USA) in TBS-T. The epitope of C219 has been mapped to the amino acid sequences VQEALD and VQAALD in the C-terminal and N-terminal halves of P-glycoprotein, respectively [29]. Proteins were visualized using enhanced chemiluminescence according to the manufacturer’s instructions (Pierce, Rockford, IL, USA). Images were captured by a Bio-Rad Gel Doc XR imaging system (Bio-Rad, Hercules, CA, USA) using the Manufacturer’s Quantity One software (version 4.6). MRP1 immunoblotting studies were conducted in a similar manner except that crude membrane samples were separated on NuPage 4-12% Bis-Tris gels (Invitrogen, Grand Island, NY, USA), and resulting membranes were probed first with the MRP1 antibody MRPr1 (1:100), followed by an anti-rat secondary (1:5,000). The MRP1 mAb MRPr1 was raised against a bacterial fusion protein containing amino acids 194 to 360 of human MRP1 [30] and its epitope was subsequently localized to amino acids 238 to 247 [31]. Equivalent protein loading of all gels was verified using GAPDH as a loading control.

### Data analysis

[^3^H]saquinavir accumulation values are expressed as pmol/mg protein and are presented as mean ± standard error (SE) from a minimum of three separate experiments. In an individual experiment, each data point represents a minimum of triplicate trials. For multiple comparisons, the test of repeated measures of analysis of variance (ANOVA) and the Bonferroni *post hoc* analysis was used. A value of *P* < 0.05 was considered statistically significant.

## Results

### [^3^H]saquinavir accumulation in HAPI microglia is P-glycoprotein dependent

**Figure 1:**
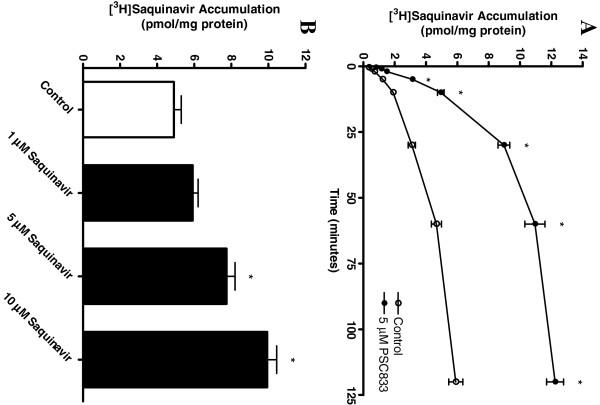
**Accumulation of saquinavir by HAPI microglia in the presence of the P-glycoprotein inhibitor PSC833. (A)** [^3^H]saquinavir (50 nM) accumulation by HAPI cells was measured in the presence (closed circles) or absence (open circles) of PSC833 (5 μM) at 0.5, 1, 2, 5, 10, 30, 60 and 120 minutes, at 37°C. **(B)** [^3^H]saquinavir (50 nM) accumulation by HAPI microglia increased significantly, in a dose dependent manner, in the presence of excess unlabelled saquinavir (1 to 10 μM). Each data point represents mean ± SE of at least three separate experiments with three replicates included per experiment; *P < 0.05, significantly different from control.

Accumulation of 50 nM [^3^H]saquinavir by HAPI microglia was initially rapid, reaching steady-state within 60 minutes (Figure 1A). All subsequent transport studies were performed at this time point. The potent and specific P-glycoprotein inhibitor PSC833 (5 μM), increased accumulation significantly, and this increase was seen at times as early as 30 seconds. This result is consistent with P-glycoprotein mediating saquinavir efflux from the cells. Addition of excess cold saquinavir to the transport buffer also increased [^3^H]saquinavir accumulation, significantly suggesting saturation of transport (Figure 1B). Similar results were previously reported for P-glycoprotein in another cultured microglia cell line, MLS-9 [11,15].

### LPS reduces [^3^H]saquinavir accumulation

**Figure 2:**
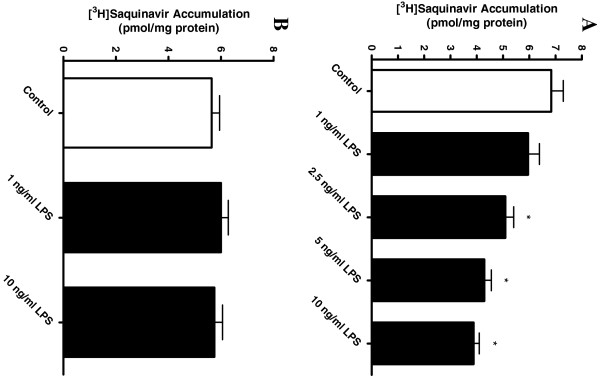
**Effect of LPS on [**^**3**^**H]saquinavir accumulation and inflammatory mediator release by HAPI microglia cells. (A)** Following 24 hours of incubation at increasing LPS concentrations (1 to 10 ng/ml) (black bars), [^3^H]saquinavir (50 nM) accumulation in HAPI cells was determined at one hour (37°C) using scintillation counting. The accumulation was dose-dependent at concentrations higher than 2.5 ng/ml LPS. **(B)** Accumulation of [^3^H]saquinavir (50 nM) for one hour following addition of LPS into the transport buffer. Each data point represents mean ± SE of at least three separate experiments with three replicates included per experiment; *P < 0.05, significantly different from control.

Exposing HAPI cells to 10 ng/ml LPS for six hours significantly reduced saquinavir accumulation by 25 ± 2.5% (*P* < 0.05). With 24 hours of exposure, 1 to 10 ng/ml LPS reduced accumulation in a concentration-dependent manner, with 10 ng/ml reducing accumulation by 43% (Figure 2A). LPS did not directly affect saquinavir accumulation, since including 1 or 10 ng/ml LPS in the transport medium did not affect saquinavir accumulation during the 60 minute assay (Figure 2B). Furthermore, viability of the cells (expressed as percent untreated control) following 24 hours of incubation with 10 ng/ml LPS (92 ± 6%) was not significantly different from that of untreated cells (100 ± 3%).

**Figure 3:**
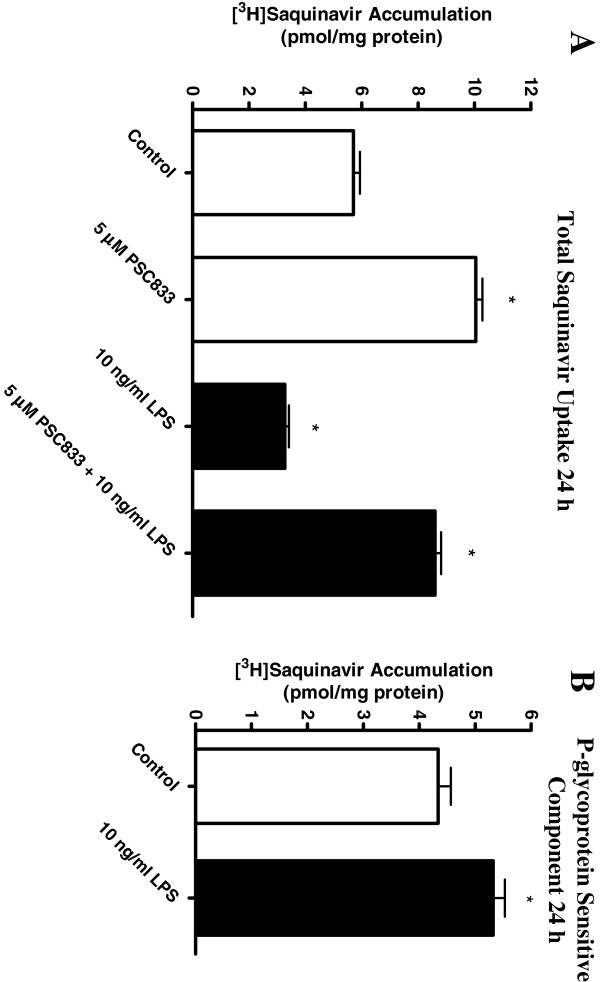
**Interactions with P-glycoprotein in HAPI microglia only partially explain the LPS-mediated changes in saquinavir accumulation. (A)** Following 24 hours LPS incubation (10 ng/ml), accumulation of [^3^H]saquinavir (50 μM) at one hour (37°C) was measured in the presence and absence of the P-glycoprotein inhibitor PSC833 (5 μM). **(B)** By subtracting the LPS-untreated values (control and PSC833 inhibited; white bars) from the LPS-treated cells (LPS control and PSC833 inhibited; black bars) the P-glycoprotein specific/sensitive component of transport was determined. Each data point represents mean ± SE of at least three separate experiments with three replicates included per experiment; *P < 0.05, significantly different from LPS-untreated control cells.

**Figure 4:**
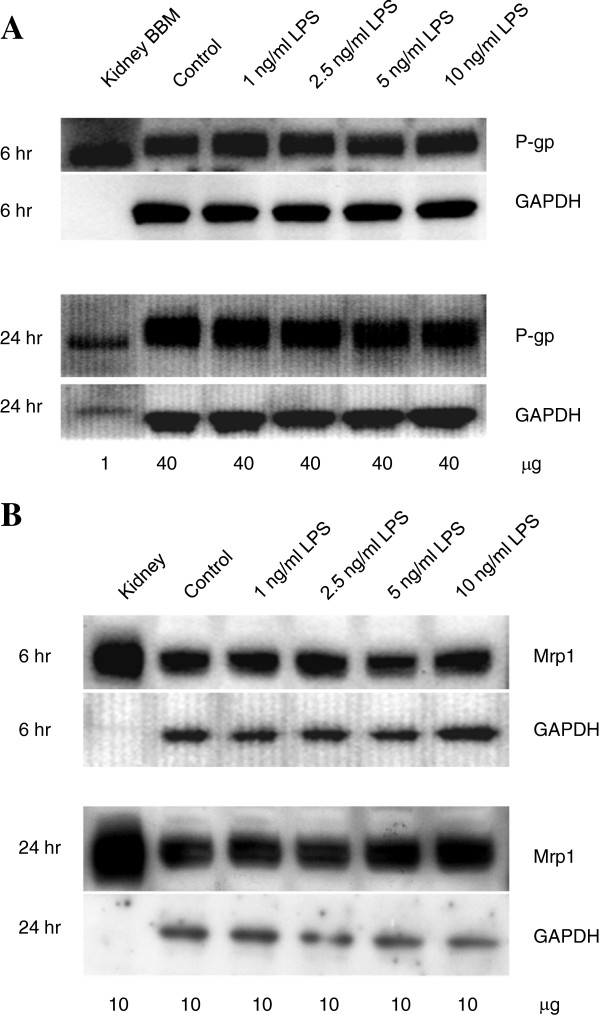
**Protein expression of P-glycoprotein and Mrp1 in HAPI cells following 6 or 24 hour LPS incubation. (A)** HAPI microglia were incubated for 6 or 24 hours in the presence of increasing concentrations of LPS (1 to 10 ng/ml). At the end of the incubation period, crude membrane fractions from the cell lysates or from rat kidney brush border membranes (positive P-glycoprotein control) were prepared as indicated in the text, and immunoblotting of P-glycoprotein was performed. The proteins (1 or 40 μg each) were separated on NuPage 7% sodium-acetate gels, transferred to polyvinylidene difluoride membranes electrophoretically and incubated with a P-glycoprotein monoclonal antibody, C219 (1:100), followed by an anti-mouse HRPO-secondary (1:1,000). **(B)** Immunoblotting of Mrp1 in HAPI microglia treated for 6 or 24 hours with increasing concentrations of LPS (1 to 10 ng/ml) was undertaken in a similar manner to P-glycoprotein except that the proteins (10 μg) were separated on NuPage 4 to 12% Bis-Tris gels, and incubated with a Mrp1 monoclonal antibody, MRPr1 (1:100), followed by an anti-rat HRPO-secondary (1:1,000). Whole rat kidney lysate (10 μg) was used as a positive Mrp1 control. Representative immunoblots are shown.

To determine whether the change in saquinavir accumulation with LPS exposure was due to altered P-glycoprotein function, we measured drug accumulation in the presence and absence of 5 μM PSC833 in control and LPS-treated (24 hours) cells. As expected, total saquinavir accumulation decreased in the presence of 10 ng/ml LPS and increased in the presence of PSC833 (5 μM) in both control cells (+77%) and in LPS-exposed cells (+58%; Figure 3A). The PSC833-sensitive component of saquinavir accumulation increased significantly in the LPS-treated cells (+31%), suggesting that increased P-glycoprotein mediated transport (Figure 3B). We found a similar trend in cells exposed to 10 ng/ml LPS for six hours (data not shown). Importantly, following exposure to 1 to 10 ng/ml LPS, we observed no changes in P-glycoprotein expression at the protein level (Figure 4A).

### Other transporters do not contribute to decreased saquinavir accumulation

We previously demonstrated that saquinavir interacts with a second efflux transporter in microglia, namely Mrp1 [14]. We used the Mrp inhibitor MK571 to measure the Mrp-mediated component of transport in the HAPI cells. In contrast to P-glycoprotein, there was no significant change in the Mrp sensitive transport component in HAPI microglia following LPS exposure for six hours (−9 ± 3%) or 24 hours (−14 ± 14%). Protein expression (Figure 4B) was also unchanged at these time points.

In addition to P-glycoprotein and multiple MRP isoforms, saquinavir and other AR compounds interact with multiple members of the solute carrier (SLC) transporter family, including the human organic anion polypeptide (OATP) transporters OATP1B1, 1B3 and 2B1 [32,33], and the human organic cation transporters OCT1 and 2 [34]. At present, the expression and function of SLC transporters in microglia is unknown. We determined whether expression of well-characterized anionic and cationic SLC transporters could be detected in HAPI microglia at the transcriptional level. Using RT-PCR, we could not detect transcripts of Slco1a1, 1a2, or 1a5, which encode protein for Oatp1a1, 1a2 and -1a5, respectively. Slc22a6, 22a8 and 22a1 genes which encode for Oat1, 3, and Oct1, respectively, were also undetected in HAPI cells. The Slc22a2 gene transcript encoding for Oct2 was detected in HAPI cells, but was unchanged in the presence of 10 ng/ml LPS (data not shown).

### Multiple molecular pathways regulate P-glycoprotein in HAPI microglia exposed to LPS

**Figure 5:**
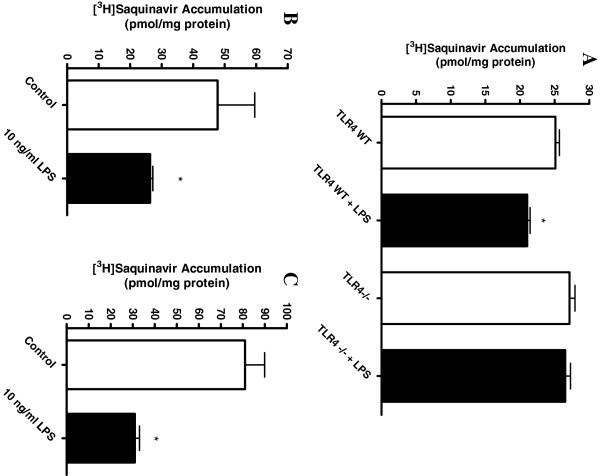
**Decreased saquinavir accumulation by HAPI microglia in the presence of LPS is attenuated in TLR4 deficient mice. (A)** Primary microglia were isolated from TLR wild-type (TLR4 WT) and TLR4 deficient (TLR4^−/−^) mice, as described. In primary microglia isolated from TLR4 WT mice, incubation with 10 ng/ml LPS for 24 hours decreased saquinavir significantly, that is, by approximately 16%. This effect was attenuated in microglia derived from the TLR4^−/−^ deficient mice, where there was no difference between LPS-treated and untreated deficient mice or the untreated WT mice. *P < 0.05, significantly different from untreated TLR WT controls. **(B)** Primary microglia derived from Wistar, and **(C)** Fisher rats incubated with 10 ng/ml LPS for 24 hours at 37°C also showed a significant decrease in saquinavir accumulation at one hour. *P < 0.05, significantly different from untreated control cells.

Exposure of microglia to LPS produces a robust pro-inflammatory response, including the production and release of cytokines, chemokines, reactive oxygen species and other pro-inflammatory mediators. This response is largely mediated through multiple cell surface receptors including TLR-2, TLR-4 and multiple scavenger receptors [35]. The released inflammatory mediators can then interact with additional cell surface receptors and intracellular pathways, initiating new molecular cascades and inciting a self-propelling cycle of cellular activation. Pre-treatment of HAPI microglia with inhibitors of scavenger receptors and NADPH oxidase (that is, fucoidan and DPI, respectively) did not attenuate the LPS-related decrease in saquinavir accumulation mediated by LPS (Table 2). However, decreases in saquinavir accumulation by HAPI microglia were partially attenuated by antibodies to TLR2 and TLR4 (Table 2). To confirm that LPS effects were mediated by TLR-4, we used primary cultures of microglia from wild-type and TLR4 deficient mice. In wild-type cultures, exposure to 10 ng/ml LPS significantly decreased saquinavir accumulation (Figure 5A). However, this decrease was small, averaging only 16% of total accumulation. Importantly, in microglia from TLR-4 deficient mice, LPS exposure did not alter saquinavir accumulation. We repeated the basic LPS exposure experiment in primary microglia from Wistar rats and Fisher rats and found that LPS exposure reduced saquinavir accumulation by 45% and 61%, respectively (Figures 5B and C). These effects were similar to that seen in the rat-derived HAPI microglia cell line (approximately 64% of control), and considerable higher than that observed in the mouse, suggesting species differences in LPS sensitivity. Nonetheless, the decrease in saquinavir accumulation by LPS observed in the TLR4 WT mice was completely abrogated in the TLR4 deficient mice (TLR4^−/−^).

**Table 2:** **Effect of various signal transduction pathway inhibitors on [**^**3**^**H]saquinavir accumulation in microglia**

**Inhibitor**	**Concentration**	**Saquinavir transport (% control)**		
		**10 ng/ml LPS**^**a**^	**Inhibitor alone**	**LPS + Inhibitor**
Anti-TLR2	1 μg/ml	62 ± 2	99 ± 3	^b^82 ± 2
Anti-TLR4	1 μg/ml	62 ± 2	99 ± 4	^b^75 ± 3
Fucoidan	50 ng/ml	67 ± 3	92 ± 5	57 ± 2
DPI	10 nM	63 ± 2	94 ± 9	64 ± 11
Tempol	20 μM	63 ± 2	88 ± 7	60 ± 3
IL-1β RA	10 μg/ml	73 ± 2	102 ± 4	77 ± 3
Anti-IL-1β	0.5 μg/ml	63 ± 3	91 ± 2	64 ± 2
Anti-TNF-α	0.5 μg/ml	63 ± 3	89 ± 2	64 ± 2
TIMP3	10 ng/ml	57 ± 4	97 ± 5	61 ± 2
1400 W	5 μM	61 ± 3	94 ± 3	70 ± 2
L-NMMA	10 μM	58 ± 3	98 ± 7	63 ± 2
BIM	25 nM	61 ± 4	92 ± 3	67 ± 6
Wortmannin	1 μM	70 ± 2	99 ± 6	68 ± 3
SB203580	10 μM	63 ± 2	93 ± 2	63 ± 3
JNKII	10 μM	66 ± 3	96 ± 2	64 ± 3

Pathway inhibitor acronyms defined in text. ^a^*P* < 0.05 as compared to untreated controls. ^b^*P* < 0.05 as compared to LPS alone and untreated controls (partial inhibition).

**Figure 6:**
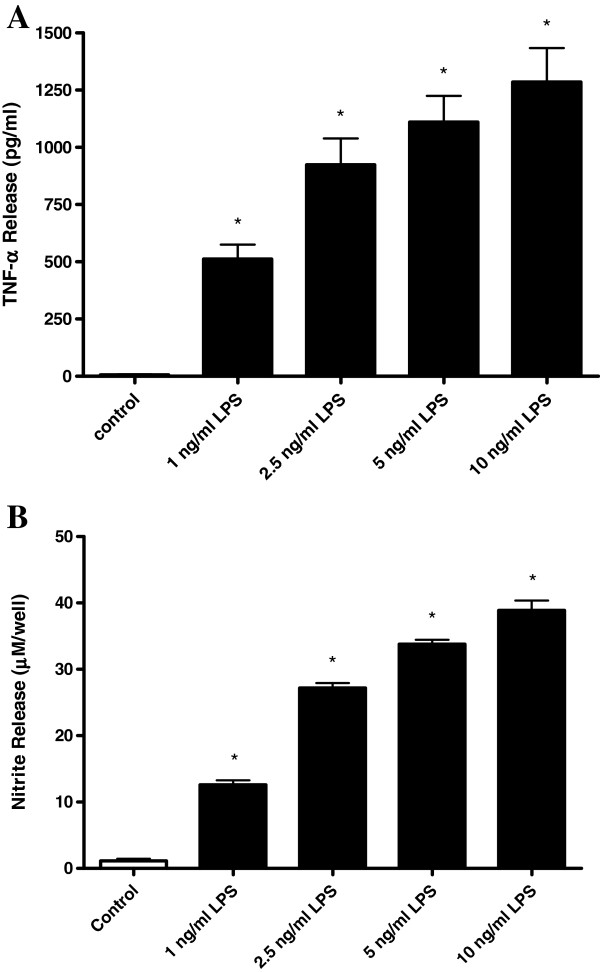
**TNF-α and nitrite release by HAPI cells following LPS exposure.** Release of TNF-α **(A)** and nitrite **(B)** by HAPI cells ino the medium was measured following 24 hours LPS incubation at 1 to 10 ng/ml. Each data point represents mean ± SE of at least three separate experiments with three replicates included per experiment; *P < 0.05, significantly different from untreated control (open bars).

Following LPS exposure, primary microglia extrude pro-inflammatory mediators such as TNF-α, IL-1β and NO [35]. Following 24 hours exposure to LPS, HAPI microglia showed a concentration-dependent increase in cellular extrusion of TNF-α (Figure 6A) and NO (Figure 6B). Interestingly, exposure of HAPI microglia to exogenously applied TNF-α and IL-1β, or NO generated by the use of the NO donor DEA NONOate did not alter saquinavir accumulation (Table 3). Pre-incubation of HAPI with inhibitors targeted against the cytokines themselves (anti-TNF-α, anti-IL-1β), or molecular pathways involved in up- or downstream signaling events for the cytokines (IL-1β receptor antagonist, TIMP3) or NO synthetase (1400W and L-NMMA) also did not alter the ability of the cells to accumulate saquinavir (Table 2).

**Table 3:** **Effect of various pathway activators on [**^**3**^**H]saquinavir accumulation in microglia**

**Activator**	**Concentration**	**Saquinavir transport (% control)**	
		**10 ng/ml LPS**^**a**^	**Activator alone**
TNF-α	10 ng/ml	73 ± 2	98 ± 6
IL-1β	10 ng/ml	77 ± 2	99 ± 2
DEA NONOate	1 μM	61 ± 2	92 ± 5
DEA NONOate	10 μM	61 ± 2	92 ± 4
DEA NONOate	25 μM	61 ± 2	99 ± 6
ET-1	100 nM	61 ± 3	95 ± 2
PMA	1 μM	58 ± 2	94 ± 4
PCN	1 μM	54 ± 2	99 ± 4
PGE_2_	1 μM	61 ± 3	103 ± 4

DEA NONOate, 2-(*N*,*N*-dimethylamino)-diazenolate-2-oxide; ET-1, endothelin-1; IL-1β, interleukin-1-beta; TNF-α, tumor necrosis factor-alpha; PMA, phorbol-12-myristate-13-acetate; PCN, 5-pregnen-3β-ol-20-one-16α-carbonitrile; PGE_2,_ prostaglandin E2. ^a^In each separate experiment, 10 ng/ml LPS decreased saquinavir accumulation significantly (^a^*P* < 0.05, statistically significant from untreated controls).

**Figure 7:**
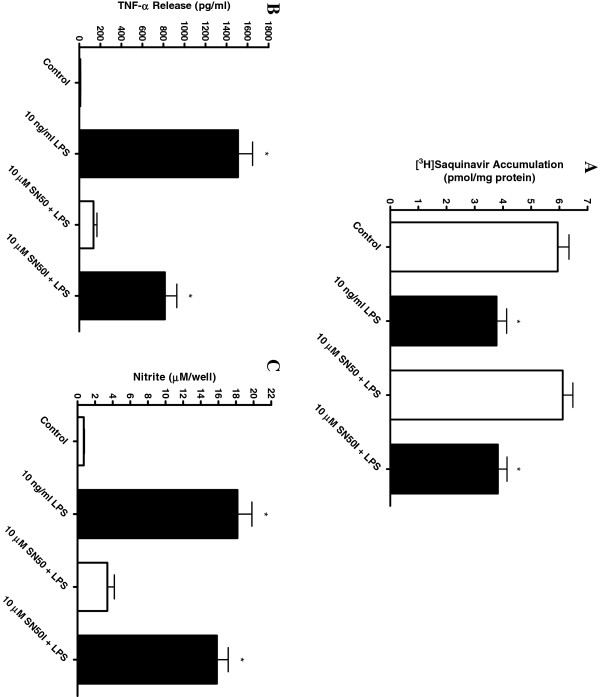
**Saquinavir accumulation and inflammatory mediator release following incubation of HAPI cells with a cell permeable NF-κβ inhibitor. (A)** Accumulation of [^3^H]saquinavir (50 nM) at one hour (37°C) following 24 hours incubation with 10 ng/ml LPS and the NF-κβ inhibitor SN50 (10 μM) or inactive control peptide SN50I (10 μM). Release of TNF-α **(B)** and nitrite **(C)** into the medium following 24 hours incubation with 10 ng/ml LPS in the presence of the NF-κβ inhibitor SN50 (10 μM) or control peptide SN50I (10 μM) was also measured under the same experimental conditions. Each data point represents mean ± SE of at least three separate experiments with three replicates included per experiment; *P < 0.05, significantly different from untreated control cells; #*P* < 0.05, significantly different from 10 ng/ml LPS and 10 μM inactive SN50I + LPS.

**Figure 8:**
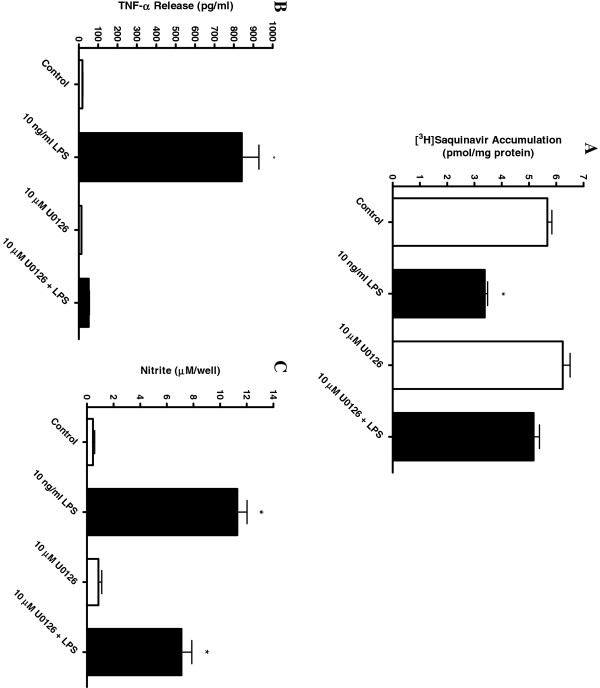
**Effect of MEK1/2 inhibitor on saquinavir accumulation, TNF-α and nitrite release.** Saquinavir accumulation and inflammatory mediator release was determined following incubation of HAPI cells with a cell permeable MEK1/2 inhibitor U0126 **(A)**. Accumulation of [^3^H]saquinavir (50 nM) in the presence and absence of 10 ng/ml LPS and 10 μM U0126 at one hour at 37°C. Release of TNF-α **(B)** and nitrite **(C)** into the medium following 24 hour incubation with 10 ng/ml LPS in the presence of U0126. Each data point represents mean ± SE of at least three separate experiments with three replicates included per experiment; *P < 0.05, significantly different from untreated control cells.

We further screened HAPI cells directly with a number of other well-characterized inflammatory mediators known to be involved in microglial signaling ( [36-38]) including the rat nuclear receptor PXR activator PCN (1 μM), the thromboxane A_2_ activator ET-1 (1 μM), adenylate cyclase regulator PGE_2_ (1 μM), and the protein kinase C activator PMA (1 μM) [Table 3]. None of these activators affected saquinavir accumulation. In addition, cell permeable chemical inhibitors known to specifically inhibit intracellular molecular pathways that function within microglia such as multiple kinase pathways ( [36-39]) were also tested. Full inhibition of the LPS-induced decrease in saquinavir accumulation was found for only two of the compounds tested. First, the NF-κβ translocation inhibitor SN50 fully blocked the decreased saquinavir accumulation mediated by LPS (Figure 7A). No effect was observed with an inactive SN50 control peptide, SN50I. SN50 also significantly lowered levels of TNF-α (Figure 7B) and nitrite (Figure 7C) release in response to LPS, confirming a generalized decrease in microglial activation. Second, the MEK1/2 inhibitor U0126 also fully blocked the LPS-mediated effects on saquinavir accumulation by the cells (Figure 8A and TNF-α (Figure 8B) and nitrite (Figure 8C) release.

## Conclusions

Here, we investigated how LPS-induced inflammation alters the function of drug transporters in microglia, the primary CNS target of HIV, using a clinically relevant concentration (50 nM) of the antiretroviral medication saquinavir as a prototypical probe substrate and the following model systems: a rat microglia cell line, HAPI, and primary cultures of rat and mouse microglia. Furthermore, we examined at a molecular level, what mechanisms may drive the observed changes in saquinavir accumulation and retention by microglia following an inflammatory LPS challenge. As noted in another rat microglia cell line [14], accumulation of saquinavir into HAPI microglia cells was rapid, reached a plateau by one hour, and was increased significantly by a potent P-glycoprotein inhibitor PSC833. In this model, an increase in saquinavir accumulation in the presence of PSC833 provides an indirect measure of compound efflux by the transporter. Following both short (6 hour) and long term (24 hour) LPS exposure in microglia, the overall accumulation of saquinavir decreased in a dose-dependent manner, with significant decreases observed at 24 hours at doses greater than 2.5 ng/ml LPS. Using LPS in the presence and absence of the P-glycoprotein inhibitor PSC833, the decrease in saquinavir accumulation was only partially explained by increases in P-glycoprotein function, that is, by increased P-glycoprotein-mediated efflux of compound from the intracellular compartment to the outside of the cell. The remainder of the unaccounted saquinavir transport surprisingly could not be explained by increases in efflux or protein expression of Mrp1, a transporter known to handle saquinavir efficiently. Although less likely, a decrease in potential uptake of saquinavir into the cells via decreased SLC uptake transporter expression/function was also considered. Transcripts of seven well- characterized SLC transporters, some already well known to interact with ARs, were examined in the presence and absence of LPS. With the exception of Slc22a2, none of these transporters (at the transcriptional level) were expressed significantly in HAPI microglia. Furthermore, Slc22a2 transcript levels in HAPI microglia were unchanged following LPS exposure. Therefore, it is unlikely that a change in SLC uptake transporters explains the reduced accumulation of saquinavir following LPS treatment.

While it was clear that LPS exposure decreased accumulation of saquinavir significantly in microglia, at least partially through a P-glycoprotein pathway, protein levels of that transporter were unchanged. One explanation for this may be that transporter function was altered by signaling pathways downstream of TLR4, resulting in for example, transporter dephosphorylation, deglycosylation, or tyrosine nitration. Indeed, activation of NF-κβ in HT29 colon cancer cells decreases transport function of another drug transporter, human MRP3, via tyrosine nitration of the protein [40]. This suggests that TLR4 signaling regulates microglial P-glycoprotein activity to some extent, which is consistent with the fact that cytokines and NO are produced 6 to 24 hours later in the microglial response to LPS but fail to impact P-glycoprotein function/saquinavir accumulation in the current study. The role of TLR4 in P-glycoprotein regulation is particularly relevant to pharmacotherapy in HIV, as there is increasing evidence that HIV proteins may activate macrophages through a TLR4 dependent pathway. In fact, a recent study shows that HIV1-Vpr induces cytokine production from macrophages through TLR4/MyD88 [41].

A second explanation for the discrepancy between altered P-glycoprotein function and expression following LPS treatment is altered trafficking of P-glycoprotein from intracellular stores to the cell surface. To actively efflux compounds, P-glycoprotein must be correctly orientated on the plasma membrane. In polarized cells such as brain capillary endothelium and choroid plexus epithelia, proper routing of intracellular reserves of transporter protein to the plasma membrane on the apical side is achieved through a series of complex molecular signaling events. In brain capillaries, intracellular stores of P-glycoprotein may cycle into and out of the endothelial membranes following exposure to proinflammatory mediators as a short-term adaptive compensation mechanism to cellular stresses [42]. Mechanisms contributing to trafficking of drug transporter proteins within microglia have not been identified. However, immunohistochemical studies of P-glycoprotein in microglia have localized the protein to both the plasma and nuclear membranes, demonstrating that intracellular compartments for the protein do indeed exist [11,43,44] and might be recruited in response to cellular stress.

The interaction of LPS with microglia at the molecular level and subsequent signaling pathway activation have been well described elsewhere [1]. At the cell surface level, LPS activation of TLR4, scavenger receptors and NADPH oxidase have all been implicated as initial events that initiate downstream intracellular signaling changes in microglia. Inhibition of the scavenger receptors (by fucoidan) and NADPH oxidase (by DPI) in the present studies did not attenuate the decrease in saquinavir accumulation following LPS challenge, whereas a TLR-4 neutralizing antibody caused partial attenuation. By decreasing TLR4 activity to a large extent using microglia from TLR4 deficient mice, full attenuation of the changes in saquinavir transport in the presence of LPS in primary microglia was seen. This demonstrates that TLR4 signaling at the cell surface is sufficient to initiate a signaling cascade that affects P-glycoprotein downstream.

In microglia, surface engagement of TLR4 by LPS leads to activation of multiple intracellular pathways including those connected to NF-κβ, AP-1, JAK/STAT, and multiple protein kinase pathways. Recent studies by Gibson *et al*., [45] have shown a role for NF-β in the regulation of P-gp in a mouse microglia cell line, BV-2. Interestingly, in this study, LPS at doses of 1 to 500 ng/ml for 12 hours reduced P-gp expression (mRNA and protein), and function using the fluorescent P-gp probe rhodamine 123. In the present study using primary cultures of mouse microglia, 10 ng/ml LPS decreased saquinavir accumulation significantly at 6 and 24 hours, presumably due to increased saquinavir efflux. The observed decrease in saquinavir accumulation in the mouse cultures was, however, modest compared to primary rat cultures, suggesting potential species differences. Whether species differences in molecular mechanisms or specific substrate handling can explain these discrepancies, remains to be confirmed.

Of all the molecular pathways examined in the present study, only inhibition of NF-κβ and MEK1/2 reversed the changes in saquinavir accumulation in microglia following LPS exposure. Given that several pro-inflammatory factors that are known activators of NF-κβ (for example, TNF-α and IL-1β) were shown to have no effect, these findings support that NF-κβ is necessary, but not sufficient to change saquinavir accumulation. These results are in stark contrast to findings in freshly isolated rat brain capillaries where LPS also initiates activation of TLR4, which downstream is connected to alterations in TNF-α, ET-1, iNOS and PKC activation, and ultimately results in increased P-glycoprotein protein expression and consequently function in the capillaries [42]. This may not be surprising, as the transporter profile in glial cells is quite different compared to cells of the BBB. Most notably, cultured microglia do not express significant levels of Mrp2 [12], Bcrp [46] or mRNA of any of the important SLC uptake transporters (that is, Slco221a2, 1a5, and Slc22a8) expressed at the BBB. Given the redundant nature of the LPS response in microglia (that is, multiple pathways are initiated via multiple cell surface receptors), we cannot rule out the possibility that compensatory pathways mask the effects of inhibition or activation of a single pathway in our cell cultures. Further investigations *in vivo* using knockdown strategies may be helpful to fully elucidate all the pathways that are involved.

In summary, we have demonstrated that exposing microglial cells to LPS decreases cellular accumulation of one representative antiretroviral medication. The ability of LPS to significantly decrease saquinavir accumulation was consistent between microglia derived from multiple species (rats versus mice), multiple strains within the same species (Wistar versus Fisher rats), and multiple cell preparations (cultured cell line versus primary cells). Using PSC833, a non-immunosuppressive cyclosporine-A analog and potent P-glycoprotein inhibitor, the decrease in saquinavir accumulation in cultured microglia was consistent, in part, with an increase in P-glycoprotein-mediated drug efflux. This increase in transporter activity and its absence in cells from TLR4-deficient mice suggest an important role for TLR4 in microglial P-glycoprotein function and demonstrate its importance for HIV pharmacotherapy. These results confirm that the presence of neuroinflammation within the brain parenchymal compartment can further exacerbate the ability of glial cells to actively extrude antiretroviral agents, and explains in part why treatment of neurologically-based HIV strains remains difficult despite our best efforts.

## Abbreviations

ABC: ATP-binding cassette; AR: Antiretroviral; BBB: Blood-brain barrier; bp: Base pairs; CNS: Central nervous system; EBSS: Earle’s balanced salt solution; HIV-1: Human immunodeficiency virus type 1; LPS: Lipopolysaccharide; MRP: Multidrug resistance protein; RT-PCR: Reverse transcriptase-polymerase chain reaction.

## Competing interests

The authors declare they have no competing interests.

## Authors’ contributions

SD conceived the studies, carried out transport, western blot and biochemical assays, isolated primary microglia, wrote the draft and approved manuscript. MLB isolated primary microglia, carried out biochemical assays, contributed to the draft and approved manuscript. DMT conceived and carried out RT-PCR studies and approved the manuscript. MGB conceived the biochemical assays, helped draft the manuscript and approved the manuscript. PTR carried out western blot studies, contributed to the draft manuscript and approved the manuscript. RB contributed to design of studies, helped draft the manuscript and approved the manuscript. DSM conceived studies, contributed to design of studies, helped draft the manuscript and approved the manuscript. All authors read and approved the final manuscript.
